# Behavioural Disturbances in a Temperate Fish Exposed to Sustained High-CO_2_ Levels

**DOI:** 10.1371/journal.pone.0065825

**Published:** 2013-06-04

**Authors:** Fredrik Jutfelt, Karine Bresolin de Souza, Amandine Vuylsteke, Joachim Sturve

**Affiliations:** Department of Biological and Environmental Sciences, University of Gothenburg, Gothenburg, Sweden; Université Pierre et Marie Curie, France

## Abstract

As atmospheric CO_2_ levels rise, the CO_2_ concentration in ocean surface waters increases through a process commonly referred to as ocean acidification. Recently, surprising behavioural modifications has been detected in the early life stages of tropical coral reef fish exposed to ocean acidification-relevant CO_2_ concentrations, but it has been unclear if this effect could occur in temperate waters. Here we show several severe behavioural disturbances, including effects on boldness, exploratory behaviour, lateralisation, and learning in a temperate fish, the three-spined stickleback (*Gasterosteus aculeatus*). The behavioural effects were consistent throughout the exposure period and increased in effect size with exposure time. We observed the effects on adult sticklebacks, a species known to be tolerant to other environmental stressors. Our findings suggest that behavioural abnormalities that stem from CO_2_ exposure are not restricted to sensitive tropical species or early life stages and may therefore affect fish on a global scale. The severity of disturbances and the possibility of a serious behavioural problem for fish across the globe is cause for concern.

## Introduction

Anthropogenic CO_2_ emissions are increasing the atmospheric CO_2_ concentration, which drives increasing dissolution of CO_2_ into the oceans through a process commonly referred to as ocean acidification [1]. Teleost fish have been regarded as highly tolerant to the effects of ocean acidification [2]–[4]. As fish are active organisms with a high metabolism, their muscle activity produces CO_2_ at a variable rate. Consequently, they experience internal fluctuations in CO_2_ concentration. The gill is the major organ for pH regulation where hydrogen ions are excreted and bicarbonate ions absorbed [2]. These mechanisms effectively buffer the blood pH during transient periods of high water CO_2_ concentration, and thus, fish have been assumed to be tolerant to the relatively modest CO_2_-challenge of ocean acidification [3], [5]. Maintaining blood pH despite CO_2_ exposure, however, requires modifications in concentrations of blood ions, including increased HCO_3_^−^, decreased Cl^−^, and increased Na^+^ concentrations [5]–[7].

Recently a series of studies from an Australian group reported unexpected behavioural effects in several coral reef species [8]. The effects include olfactory disturbance and olfaction preference reversal in larval clownfish [9]; impairment of visual risk assessment in juvenile damselfish (*Pomacentrus amboinensis*) [10]; reduced auditory response [11] and learning [12] in clownfish (*Amphiprion percula*); reduced olfactory prey detection, reduced feeding, and increased activity in brown dottyback (*Pseudochromis fuscus*) [13]; and reduced lateralisation and prey detection in a larval *Neopomacentrus azysron*, another coral reef species [14], [15]. Atlantic cod larvae were found to be behaviourally resistant to very high pCO_2_ (4200 µatm) [16], indicating that sensitivity to CO_2_ changes can be highly variable among teleost fish. It has been suggested that fish living at the warm edge of their tolerance range are more sensitive to CO_2_-induced acidification [17]. As a result, tropical reef fish have been suggested to be among the most sensitive.

Nilsson (2012) suggested a neural mechanism as the cause of some or all of the observed behavioural effects of CO_2_ exposure. As fish experience increased CO_2_ with accompanying acidosis, they partially reduce the acidosis via the Cl^−^/HCO_3_^−^ exchanger in the gill epithelium. This leads to decreased Cl^−^ concentration as well as increased HCO_3_^−^ concentration in the extracellular fluid. The GABA_A_ receptor is a major vertebrate inhibitory receptor [18], and its main mechanism is hyperpolarisation of the post-synaptic neuron by the function as an ion channel permitting the passage of negatively charged Cl^−^ and HCO_3_^−^ ions. Reduced extracellular Cl^−^ concentrations reduce the electrochemical gradient for Cl^−^ influx, while increased intracellular HCO_3_^−^ concentrations may lead to efflux of anions and cause depolarisation of post-synaptic neurons and therefore possibly a reversal of normal GABA_A_ receptor function [15]. The ubiquitous nature of this receptor in the vertebrate brain suggests that this mechanism likely affects many aspects of behaviour. To date, however, only one study has tested this hypothesis [15].

The three-spined stickleback is a species with remarkable physiological plasticity and high tolerance to fluctuations in water chemistry and temperature [19]. This species is found in a wide range of habitats, from marine to limnic and Arctic to subtropical waters [19]. The ability to acclimatise to such wide environmental conditions suggests that these fish, in theory, may be tolerant to the increases in pCO_2_ predicted for the end of the century.

Thus, to test the hypothesis that temperate fish are more tolerant to CO_2_ exposure than tropical reef fish we exposed sticklebacks to control water (330 µatm CO_2_) or high pCO_2_ water (991 µatm CO_2_) to mimic present-day surface CO_2_ concentrations and a predicted scenario for CO_2_ concentrations in the year 2100 (the fossil fuel intensive IPCC A1F1 emission scenario [20]), respectively. A range of behavioural tests were performed after 20 days of exposure and repeated after 40 days of exposure to test for long-term acclimation capacity. The behavioural tests were lateralisation, a measure of the behavioural asymmetry of individuals; novel object test, a widely used method for assessing boldness and curiosity in fish; and an escape chamber test designed for testing exploratory behaviour and boldness.

## Materials and Methods

### Ethics statement

Rearing, handling and experimental procedures were approved by the ethical committee on animal experiments of Gothenburg, Sweden (ethical permit: Fredrik Jutfelt 100-2010 and 151-2011).

### Experimental animals

The study was conducted at Sven Lovén Centre for Marine Sciences, University of Gothenburg, Kristineberg (Sweden) in July and August of 2012. Marine female sticklebacks (*Gasterosteus aculeatus*) were caught using a seine net in Sälvik, Fiskebäckskil bay, Lysekil, Sweden (geographic coordinates: 58°14′50″N11°27′30″O). A total of 100 fishes were distributed into ten 25 L glass aquaria (ten fish each). Five aquaria were randomly assigned “control” and five “CO_2_”. The aquaria were constantly supplied with flow-through seawater at a rate of two L min^−1^ from one of four header tanks (200 L). Each header tank had a flow of five L min^−1^ of flow-through seawater taken from five m depth, and constant aeration. The fish were kept at 14 h∶10 h light∶dark cycle and fed *ad lib* twice daily with frozen Artemia naupli. Water salinity, oxygen saturation, temperature and pCO_2_ were measured daily and alkalinity was measured weekly. Oxygen saturation remained above 90% in all measurements. The water temperature was 17.6-°C±1.2 (SD) and salinity averaged 24.2 PSU±3.4 (SD).

### Experimental treatments

The pCO_2_ of all aquaria was measured daily using direct pCO_2_ measurements with an infra-red CO_2_ probe (GMT 222, Vaisala, Finland) connected to a submerged gas-permeable silicone membrane and the air inside the membrane was circulated in a closed loop to equilibrate with dissolved pCO_2_, as described elsewhere [21]. Correct factory calibration of the probe was confirmed on several occasions during the experiment using water thoroughly bubbled with a gas mixture of 1010±10 ppm CO_2_ in air (AGA, Sweden).

The pCO_2_ of the two CO_2_ treatment header tanks was maintained at the target value of 1000 µatm using pH stat Computers (Aqua Medic, Bissendorf, Germany) connected to solenoid valves regulating administration of 100% CO_2_ gas (AGA, Sweden). The pCO_2_ of control aquaria was 333 µatm±30 SD and the pCO_2_ of the CO_2_ treatment aquaria was 991 µatm (57±SD). The variance in pCO_2_ between aquaria within each header tank system was below the detection limit (<10 µatm), while the variance between header tank systems within treatment was measurable but not significant (p = 0.29). The seawater carbonate system speciation was calculated using salinity, temperature, pCO_2_, and alkalinity in CO2calc (Hansen, USGS, USA); constants: [22] and [23]. The water chemistry data is summarized in table 1.

**Table 1:** Water chemistry for the treatments Control and Elevated CO_2_.

Parameter	Control	Elevated CO_2_
pCO_2_ (µatm)	333.0±30.0	991.3±56.6
Alkalinity (TA)	1890±386	1839±131
Salinity (PSU)	24.2±3.4	24.2±3.4
Temp (°C)	17.6±1.2	17.6±1.2
pH_tot_ (calc.)	8.08±0.085	7.65±0.031
Ωaragonite (calc.)	2.09±0.74	0.82±0.11
Ωcalcite (calc.)	3.34±1.19	1.31±0.18

Temperature, salinity, pCO_2_, and alkalinity (A_T_) are measured data; pH_tot_, Ω_aragonite_ and Ω_calcite_ are calculated data using CO2calc (USGS, USA). Data is presented as means ± SD.

Half of the fishes were exposed to increased pCO_2_ over a period of 43 days while the other half was kept in control water. Aquaria were randomly attributed control or elevated CO_2_. The fish were allowed to acclimatize to the aquaria for ten days before the CO_2_ exposure started.

### Behaviour experiments

The behavioural experiments were carried out in the same environmental conditions (temperature, salinity, light, pCO_2_) as during the exposure. 20 fishes from each treatment (randomly netted) were used in each experiment. All tests were done by direct visual observation. Experiments were performed using individual sticklebacks gently introduced into the experimental chambers, always at the same starting point and position. PlastKapTek, Sweden manufactured the acrylic experimental chambers. The behavioural tests were performed twice, on four consecutive days each time, with one behavioural test each day. The tests were performed on days 20–23 and 40–43 after initiation of exposure.

#### Lateralization

**Figure 1:**
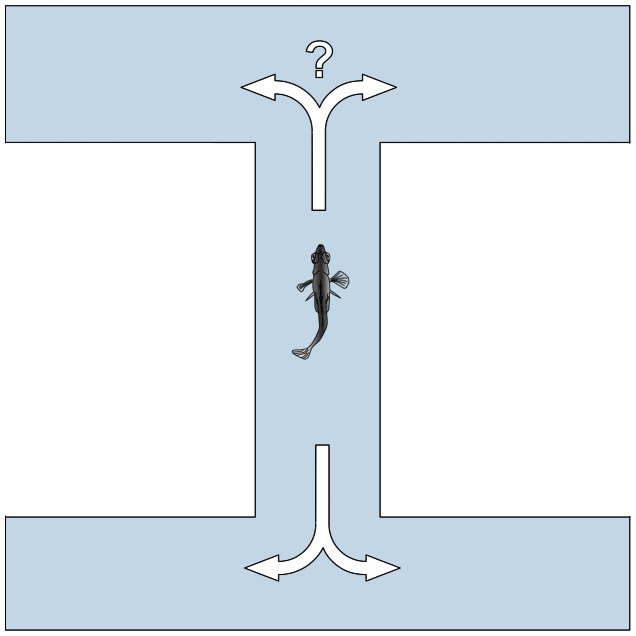
View from above of the double T-chamber used for lateralization tests. A turning choice is manually recorded every time the fish leave the central channel and enter one of the side channels. The use of a double T minimizes handling of the fish as multiple choice events can be performed by only gently encouraging the fish back along the channel.

Fishes were individually introduced into a double T-chamber (dimensions: 50 cm long with a 9 cm wide double T-channel (figure 1), according to Domenici et al. 2012) and gently encouraged by a plastic rod to move forward until a left or right turning choice was made. The procedure was repeated twelve times for each of 20 fish at day twenty, and twenty times for each of 25 fish at day 40. The purpose of increasing the number of turning events and n at day 40 was to reduce variance and increase statistical power. The relative and absolute lateralization indexes were calculated according to Domenici et al. 2012.

#### Novel object

Fishes were left to settle individually in a test aquarium for 3 min, followed by the introduction of the novel object (a 5 cm Rubik's cube) to the aquarium. The time each fish spent investigating the object was recorded during 7 minutes. The novel object test is intended to measure fear of novelty, and can thus be used to categorize fish on a bold to shy axis [24].

#### Escape challenge

Fish were placed in a white vertical cylindrical chamber (Ø16 cm×9 cm with a Ø5 cm exit hole on the side), with their heads facing away from the chamber's exit. Time spent inside the chamber until escape was recorded. The procedure was performed twice for each fish. We had not seen this experiment described elsewhere and it was designed to assess exploratory behaviour after pilot studies indicated reduced exploratory behaviour.

### Statistics

No tank effects were detected (one-way ANOVA, p>0.1), so tanks were pooled within treatment. Statistical differences between treatment groups were tested for using an independent samples *t*-test with equal variances not assumed in SPSS. Differences are considered statistically significant when p<0.05, and p-values are given in the figures. Data is presented as mean ± SEM unless otherwise noted.

## Results

**Figure 2:**
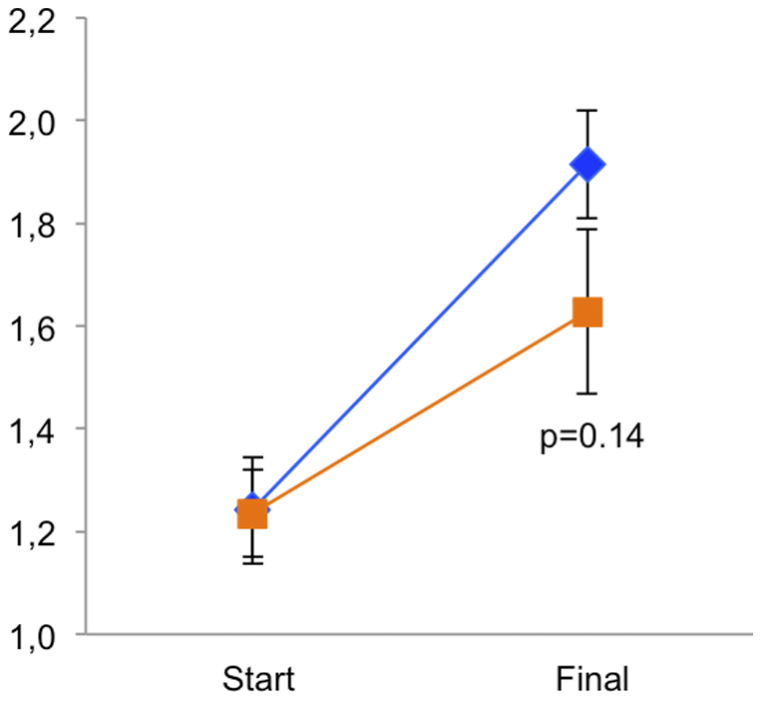
Weight at experiment start and finish (day 43). Control fish in blue and CO_2_-exposed fish in orange. N = 50 for each treatment. Data is shown as means ± SEM.

The fish gained weight during the experiment (Figure 2) and the treatment did not significantly affect final weight (p = 0.14). Total mortality over the whole experimental period was high as expected in post-spawning females: 36% in the control group and 28% in the CO_2_ group, but was not affected by CO_2_-exposure (p = 0.59).

**Figure 3:**
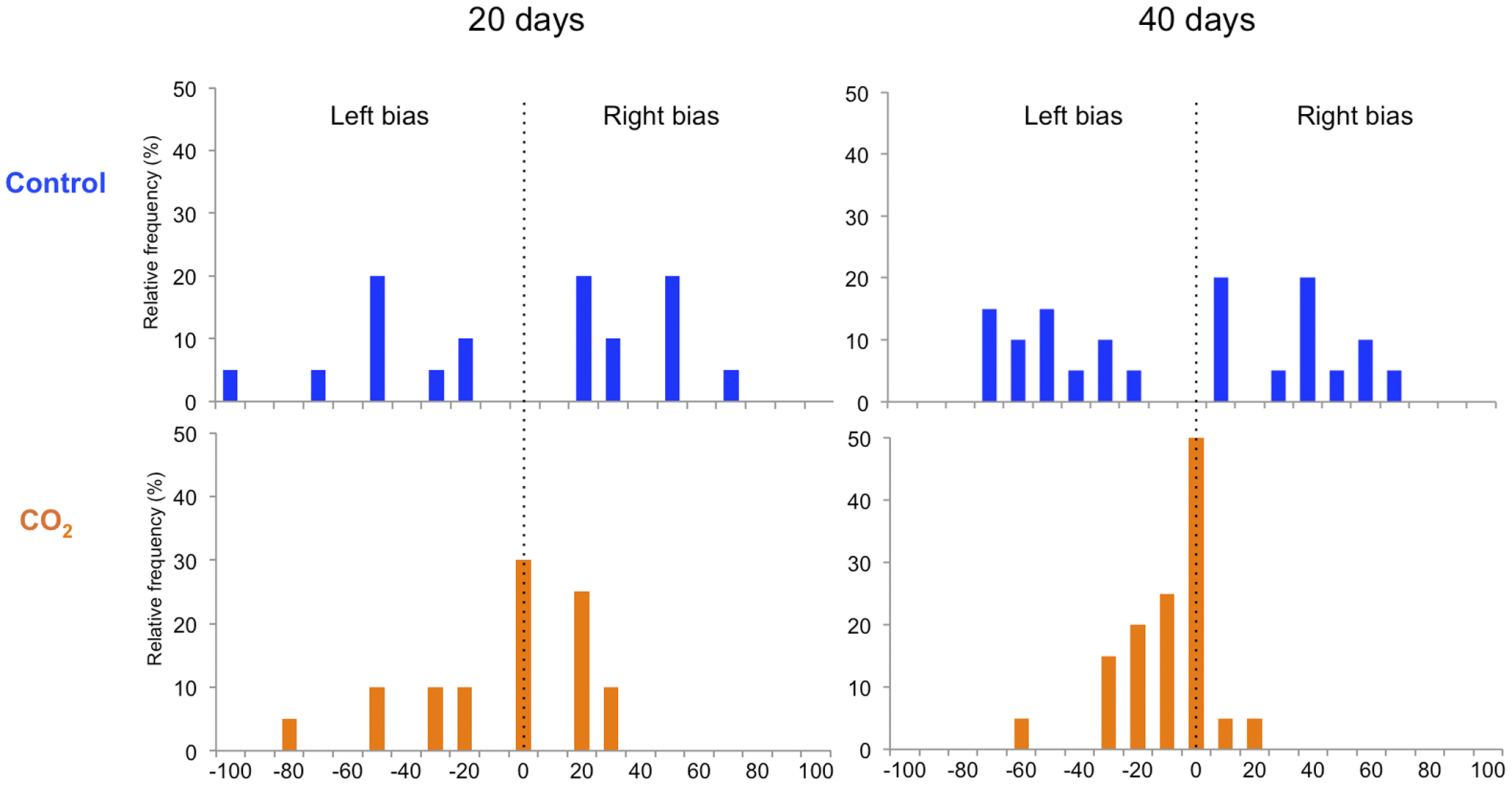
Relative lateralisation index of fish exposed to control water or CO_2_-enriched water for 20 and 40 days. The histograms show the frequency of fish with each side preference from −100 to 100, where −100 indicates that all turns were to the left, 0 indicates that half of the turns were to the left and half were to the right, and 100 indicates that all turns were to the right. N = 20–25. See figure 4 for statistical analysis.

**Figure 4:**
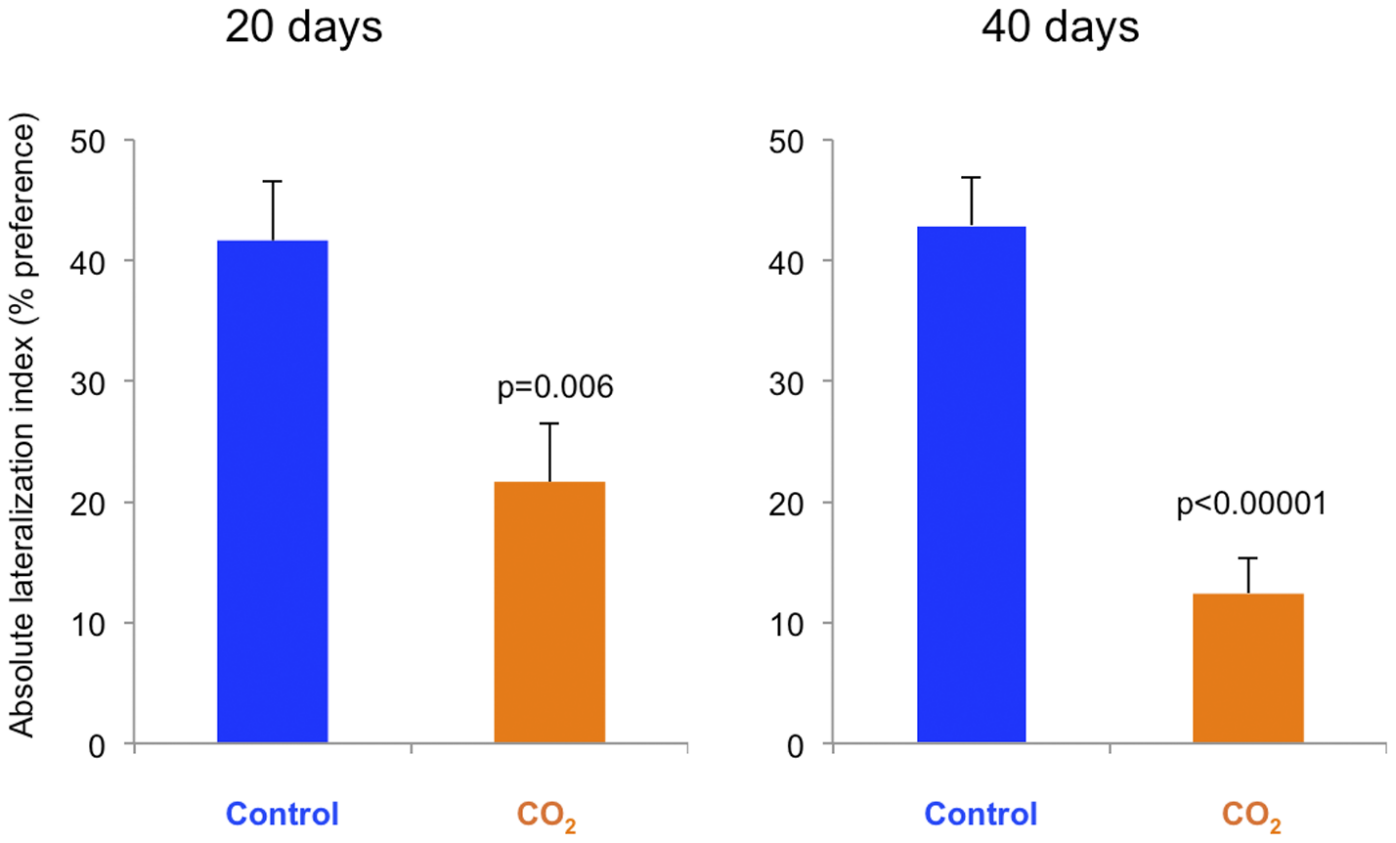
Absolute lateralisation of fish exposed to control water or CO_2_-enriched water for 20 and 40 days. N = 20–25. Data are presented as means ± standard error of the means (SEM), and *p*-values indicate statistical significance (*t*-test).

The control fish showed a wide spread of turning preferences, shown as relative lateralization index, while the CO_2_-exposed fish had a reduced distribution (figure 3). This difference is shown as absolute lateralization index (Figure 4) where the average turning preference is reduced by CO_2_-exposure. At day 20 the difference is a factor of 2 (p = 0.006), while at day 40 the effect size is larger with a factor of 3.6 (p<0.00001).

**Figure 5:**
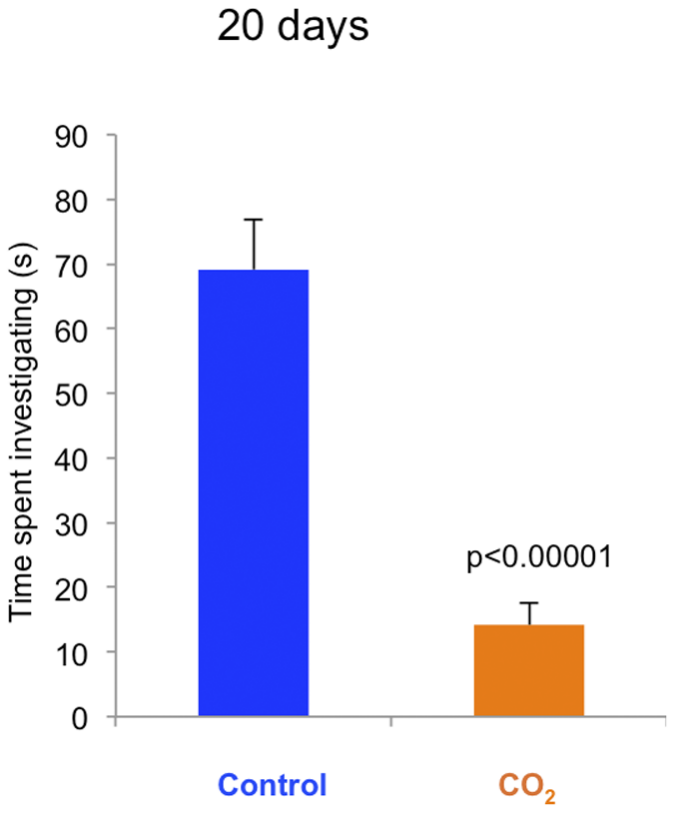
Novel object test. Bars show the average time spent investigating a novel object after 20 days of control or CO_2_ exposure. N = 20 for each treatment. Data are presented as means ± SEM, and the *p*-value indicates statistical significance (*t*-test).

When presented with a novel object control fish showed curiosity and explored the object. The average time spent investigating the object was five times longer for control fish than CO_2_-exposed fish (p<0.00001) at day 20 (figure 5). On day 40, neither group showed interest in the Rubik's cube, possibly because the fish had examined the object on the previous test occasion (data not shown).

**Figure 6:**
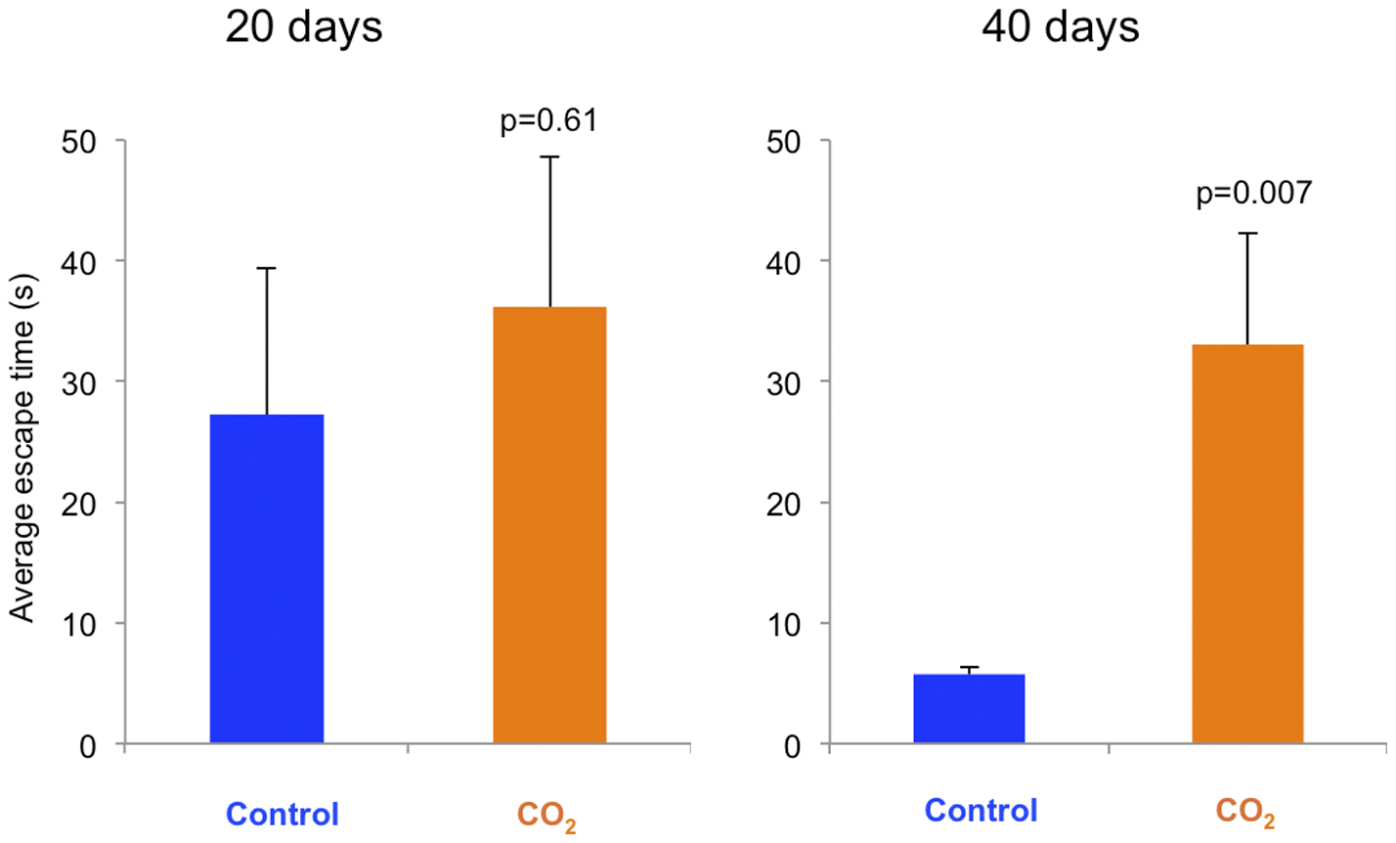
Average escape time from a chamber by fish exposed to control water or CO_2_-enriched water for 20 and 40 days. N = 20 for each treatment. Data are presented as means ± SEM, and *p*-values indicate statistical significance (*t*-test).

Both control fish and CO_2_-exposed fish took roughly half a minute to escape the escape chamber at day 20, with no significant effect of treatment (figure 6). At day 40 the CO_2_-exposed fish took the same amount of time to escape the chamber while the control fish were six times faster (p = 0.007).

## Discussion

We found surprisingly strong effects in all of the behavioural tests performed. This indicates that most, if not all, of the sticklebacks' behavioural repertoire can be affected by CO_2_.

As expected, the control fish turned to their preferred side 70% of the time on average. In contrast, CO_2_-exposed fish lacked a preference and turned to each side 50% of the time. A similar reduction in lateralisation was previously described in larvae of a coral reef fish exposed to CO_2_ [14], [15], indicating that this effect may be widespread both geographically as well as among species and life stages. A behavioural side preference is beneficial in a range of situations, such as multi-tasking, orientation, and escape [25], [26]. Any disturbance in behavioural lateralisation may therefore reduce fitness.

When fish were presented with a novel object in the form of a small Rubik's cube on day 20 of the experiment, control fish examined the object five times longer than CO_2_-exposed fish. As the novel object test is a well-established method for estimating boldness and curiosity in fish [27], [28], the finding likely indicate that CO_2_-exposed fish were less bold and/or less curious than control fish, with unknown fitness implications.

The difference in escape time at day 40 was mainly due to reduced escape time by the control group. The experiment was not specifically designed to test learning, yet the improvement by control fish in escape time from day 20 to day 40 is possibly due to memory of the escape technique from the previous trial. The CO_2_-exposed group showed no improvement at the second test, suggesting decreased learning ability. Learning is vital for acquiring new skills and behaviours. Interaction with predators is an efficient way of learning the identity of the predators, albeit sometimes with immediate costs (i.e., injury). Fish can learn to recognise predators through their own experience, through cues from injured conspecifics, and from social learning [29]. Any impairment in learning ability may therefore affect fish survival through reduced predator avoidance.

Sticklebacks are physiologically plastic with a large potential for acclimation to environmental challenges, such as changes in salinity [19] or temperature [30] and environmental toxins [31]. Acclimation to large variations in salinity and temperature commonly takes days to weeks in temperate fish [32], [33]. Additionally, *G. aculeatus* have short life spans of an average of 3 years; therefore, a 40-day exposure is considered long-term compared to the life span of sticklebacks and should be long enough to allow maximal acclimation. Because any possible acclimation failed to restore normal behaviours in the exposed fish, evolutionary selection may be required for sticklebacks to develop tolerance to high CO_2_ levels. The physiological reason behind the lack of acclimatory capacity is unknown; however, GABA_A_ receptors may have be adapted to a narrow range of ambient CO_2_ levels, whereas at higher ambient pCO_2_, the receptors are unable to function correctly due to the associated modification of extracellular and intracellular ion concentrations.

The three behavioural tests employed in this study are artificial, and so the relevance of these tests to ecological fitness is largely unknown. In addition, the CO_2_ treatment had no significant effect on survival or growth over the 40-day exposure period, suggesting that the behavioural effects are not severe enough to affect performance in an aquarium setting with *ad lib* food supply. However, because performance on all three tests, and tentatively learning, was severely degraded, it is likely that the majority of the fishes behavioural repertoire was affected by CO_2_ exposure. From a physiological perspective, ubiquitous changes in behaviour are consistent with the proposed neurophysiological mechanism involving altered GABA_A_ function [15], as GABA_A_ receptors are omnipresent in the central nervous systems of vertebrates and any disturbance to the GABA system would affect most behaviours. Thus, complex behaviours, such as prey capture, predator avoidance, and mating rituals, would likely be disturbed in a natural setting with subsequent negative fitness consequences.

## Conclusions

The current study provides indications that ocean acidification could adversely affect fish behaviour on a global scale this century. As sticklebacks are highly tolerant to many other environmental factors, it is unlikely that these animals are unusually sensitive to CO_2_ exposure. Severe behavioural effects have been documented in a few coral reef species (all from the order Perciformes) and here in temperate sticklebacks (order Gasterosteiformes). Thus, CO_2_ could potentially affect the behaviour of many, if not most, marine teleost species. As acclimation to counter the effects was not detected, despite the long exposure time, the most pressing issue now is the potential of fish to inheritably adapt to high CO_2_ levels to counter these behavioural disturbances. If the evolution of tolerance to CO_2_ is slower than the rate of CO_2_ increase, the ecological consequences of ocean acidification could be severe.
